# Effect of Litter Treatment on *Campylobacter jejuni* in Broilers and on Cecal Microbiota

**DOI:** 10.3390/pathogens9050333

**Published:** 2020-04-29

**Authors:** Amandine Thépault, Xavier Roulleau, Pauline Loiseau, Laurent Cauquil, Typhaine Poezevara, Bertrand Hyronimus, Ségolène Quesne, Florent Souchaud, Alassane Keita, Marianne Chemaly, Muriel Guyard-Nicodème

**Affiliations:** 1 ANSES, Ploufragan-Plouzané-Niort Laboratory, Hygiene and Quality of Poultry and Pork Products Unit, BP53, 22440 Ploufragan, France; amandine.thepault@anses.fr (A.T.); typhaine.poezevara@anses.fr (T.P.); segolene.quesne@anses.fr (S.Q.); florent.souchaud@anses.fr (F.S.); Marianne.CHEMALY@anses.fr (M.C.); 2 Laboratoire COBIOTEX/TERAXION, 44430 Le Loroux Bottereau, France; X.Roulleau@dietaxion.com (X.R.); b-hyronimus@cobiotex.fr (B.H.); 3 Terrena, La Noëlle, 44155 Ancenis, France; ploiseau@terrena.fr; 4 GenPhySE, Université de Toulouse, INRAE, ENVT, F-31326 Castanet Tolosan, France; laurent.cauquil@inrae.fr; 5 ANSES, Ploufragan-Plouzané-Niort Laboratory, Epidemiology, Health and Welfare Unit, BP53, 22440 Ploufragan, France; alassane.keita@anses.fr

**Keywords:** *Campylobacter*, poultry, control measure, microbiome, litter treatment

## Abstract

Since 2018, when a process hygiene criterion for *Campylobacter* in broilers at the slaughterhouse was implemented across Europe, efforts to reduce *Campylobacter* at farm level have increased. Despite numerous studies aiming to reduce *Campylobacter* colonization in broilers, no efficient control strategy has been identified so far. The present work assessed first the efficacy of a commercial litter treatment to reduce *Campylobacter* colonization in broilers during two in-vivo trials and second, its impact on cecal microbiota. The treatment does not affect broiler growth and no effect on *Campylobacter* counts was observed during the in-vivo trials. Nevertheless, cecal microbiota were affected by the treatment. Alpha and beta diversity were significantly different for the control and litter-treated groups on day 35. In addition, several taxa were identified as significantly associated with the different experimental groups. Further work is needed to find a suitable control measure combining different strategies in order to reduce *Campylobacter*.

## 1. Introduction

Campylobacteriosis has been the most frequently reported zoonotic disease every year since 2005 [1], representing an annual cost of 2.4 billion euros in the European Union [2]. Poultry constitutes both the main reservoir for this bacterium and the principal source of human contamination. In 2018, 246,571 confirmed cases were reported in the EU [1], but its incidence is grossly underestimated. In France, for example, a mean number of 4608 campylobacteriosis cases were reported annually by the National Reference Center between 2008 and 2013, but it was estimated that the multiplication factor between cases ascertained by the surveillance system and cases in the community was 115 [3]. The high community incidence of this disease leads to a high human and economic cost. Source attribution studies have identified chicken as the main host reservoir implicated in human campylobacteriosis [4,5,6] and report that the consumption of contaminated poultry is the primary source of human campylobacteriosis [7,8]. The prevalence of *Campylobacter* in broiler flocks is generally high in several European Union Member States, with more than 70% of broiler batches being contaminated; France had a high prevalence of *Campylobacter* in broiler ceca (77.2%) [9]. Moreover, the level of contamination in broiler ceca is generally high, reaching about 8 log_10_ CFU/g of cecal content [10,11]. A positive correlation is generally found between the level of contamination in the ceca and the counts on the chicken meat after slaughtering [10]. In addition, a process hygiene criterion for *Campylobacter* in broiler carcasses at the slaughtering step was implemented across Europe in 2018 [12], leading to greater efforts to reduce *Campylobacter* on poultry farms. Several control measures have been tested to reduce *Campylobacter* among colonized chickens at farm level. While several farm management practices, such as strict biosecurity and farm hygiene measures, appear to be efficient in reducing *Campylobacter* carriage in broilers flocks [13], the use of different feed additives—probiotics, organic acids or plant extracts, for example [14,15,16,17,18,19]—showed, in most cases, inconclusive results in terms of reproducibility. Another target that has not been investigated in depth so far is the litter. Litter is one of the transmission routes of *Campylobacter* in the flock [20], so litter treatments to reduce *Campylobacter* are a potential solution that should be explored. The objective of this work was to test a commercial product (COBIOTEX 410 Absorbant^®^) containing a living bacterial complex, a nutrient support and absorbent compounds acting together to limit the multiplication of pathogens. The active substance of this bacterial complex is registered as a biocide (No. 71001/040.03.001) in accordance with the European Directive 98/8/EC. Its efficacy against *Salmonella* in particular was demonstrated for registration purposes, and in this paper we describe its testing on broiler colonization by *Campylobacter*. The impact of this treatment on broilers’ cecal microbiota was also explored.

## 2. Results

### 2.1. Impact of Litter Treatment on Broilers’ Technical Performances and Their Colonization by Campylobacter

Two independent trials were carried out to test the effect of the litter treatment on cecal colonization by *Campylobacter* after an artificial challenge. During these trials, a control group reared on a non-treated litter was compared with a group reared on the treated litter; the treatment was the same in both trials, but the method used for the *Campylobacter* challenge was different.

Results showed that the treatment did not affect broiler growth, as there was no difference in mean body weight between the control and treated groups (*p* > 0.05) during the whole rearing period for both trials (Table 1).

**Figure 1:**
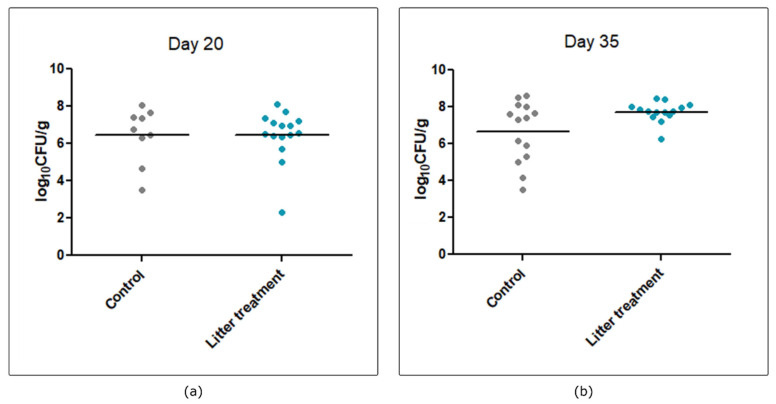
Effect of litter treatment on *Campylobacter* colonization of broiler ceca during experimental trial 1. Each bird from the control group (gray dots) and the litter-treated group (blue dots) was inoculated with *C. jejuni* C97ANSES640 at 18 days of age. (**a**) *Campylobacter* colonization on day 20; (**b**) *Campylobacter* colonization on day 35. No statistical difference was observed between control and litter-treated groups on days 21 and 35.

During both trials, chicks were free of *Campylobacter* on day 1. *Campylobacter* was not detected in the litter on day 10 (before broilers’ inoculation by *Campylobacter*) but was detected in the litter from control and litter-treated groups on days 20/21 or 35 (data not shown). During the first trial, every bird in the control and litter-treated groups was challenged with *Campylobacter*. On day 20, three days after inoculation, high levels of *Campylobacter* over 6 log_10_ CFU/g were found in broilers without any significant impact of the litter treatment (*p* > 0.05) (Figure 1). On day 35, a higher variability was observed in the control group, with *Campylobacter* loads ranging from 4.18 to 8.62 log_10_ CFU/g, whereas *Campylobacter* loads in the treated group ranged from 6.28 to 8.42 log_10_ CFU/g. However, this difference was not significant (*p* > 0.05).

**Figure 2:**
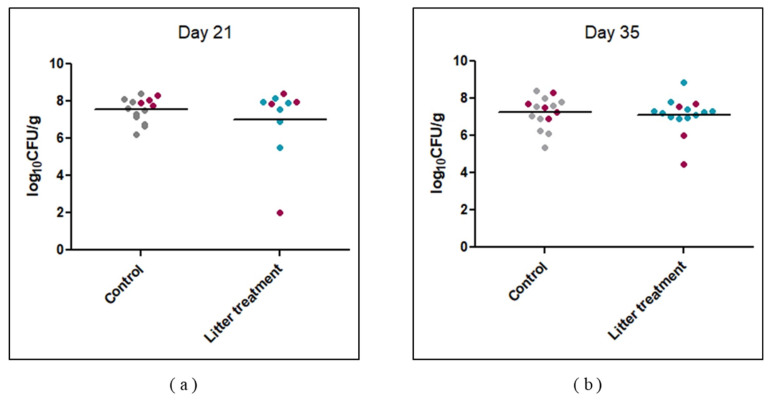
Effect of litter treatment on *Campylobacter* colonization of broiler ceca during experimental trial 2. Purple dots represent *Campylobacter* counts in broilers inoculated with *C. jejuni* C97ANSES640 at 16 days of age. Gray dots represent *Campylobacter* counts in individual cecal content from non-inoculated broilers in the control group. Blue dots represent *Campylobacter* counts in individual cecal content from non-inoculated broilers in the litter-treated group. (**a**) *Campylobacter* colonization on day 21 (**b**) *Campylobacter* colonization on day 35. No statistical difference was observed between control and litter-treated groups on days 21 and 35.

A second trial was carried out in order to test whether the treatment could reduce horizontal transmission. During this second trial, only one third of the birds were orally challenged with 4 log_10_ CFU/g of *Campylobacter*. Cecal enumeration of *Campylobacter* (Figure 2) did not reveal a significant difference between the two groups (*p* > 0.05) on day 21, five days after inoculation of only one third of the broilers. *Campylobacter* was found in them all. The same trend was observed on day 35 (Figure 2). Moreover, no statistical difference in *Campylobacter* counts was observed between inoculated and non-inoculated broilers on days 21 and 35.

### 2.2. Effect of Litter Treatment on the Composition of Broilers’ Cecal Microbiota

**Figure 3:**
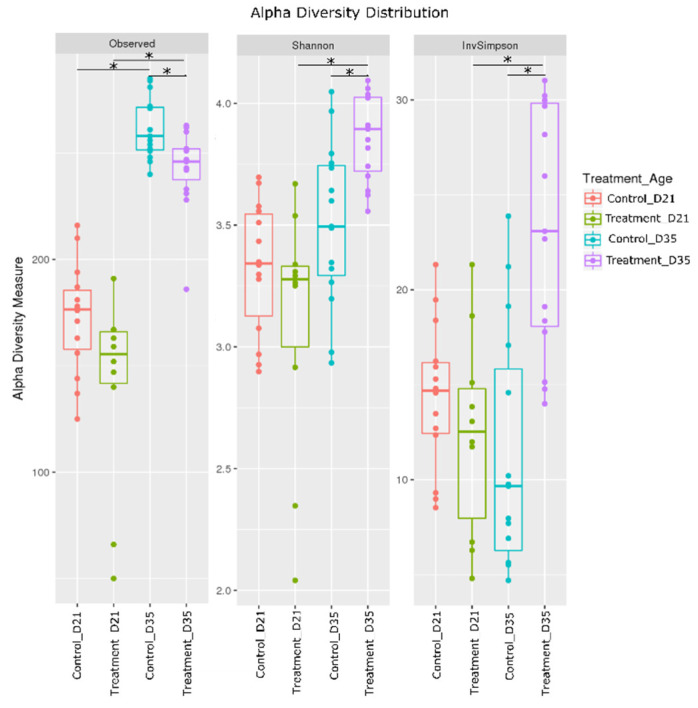
Representation of α-diversity indices for broilers’ cecal microbiota for the control and litter-treated groups on days 21 and 35. Statistical differences (*p* < 0.05) are shown by an asterisk *.

To assess the impact of litter treatment on cecal microbiota, the cecal content of the 54 broilers sampled during trial 2, and all colonized by *Campylobacter*, was analyzed using 16S metabarcoding. A total of 1,014,459 sequences were obtained from 54 samples after read demultiplexing, assembly and cleaning steps, with a median of 18,084 sequences per sample (min: 8451; max: 37,059). These sequences were then classified into 374 operational taxonomic units (OTUs) with a median of 232 OTUs per sample (min: 50; max: 285). Within samples from the control group (n = 29), medians of 177 and 258 OTUs were observed on days 21 (n = 14) and 35 (n = 15) respectively, while in the litter-treated group (n = 25), medians were 156 and 246 on days 21 (n = 10) and 35 (n = 15) respectively. No statistical difference in richness was observed between control and treated birds on day 21 (*p* > 0.05), but the litter-treated group’s microbiota on day 35 harbored less OTUs than those of the control group (*p* < 0.01) (Figure 3). Richness significantly increased (*p* < 0.0001) over time in both experimental groups (Figure 3). Shannon (evenness) and inverse Simpson indices were also calculated to assess the α-diversity within samples. Both indices increased over time only in the litter-treated group. On day 35, it was significantly higher in the group with treated litter than in the control group. These results suggest that the microbiota of broilers on treated litter contained fewer species with a more even distribution of relative abundance than in the control group, resulting in higher bacterial diversity due to the litter treatment by day 35. All the values of alpha diversity indices are shown for each cecal sample in Table S1.

**Figure 4:**
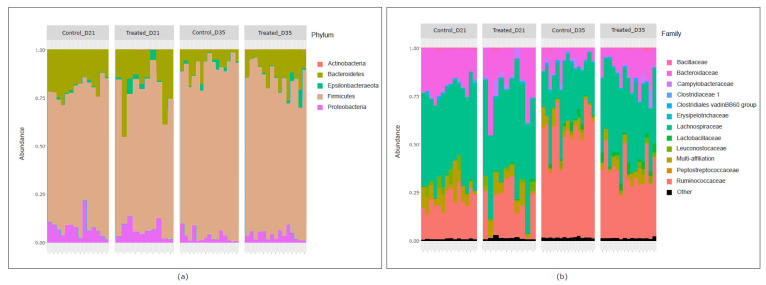
Taxonomic composition represented by relative abundance of bacterial communities from the cecal microbiota of broilers from the control or litter-treated groups on days 21 and 35. (**a**) Relative abundance of the five phyla identified in cecal microbiota represented by a bar for each sample. (**b**) Relative abundance of the 12 main families represented by a bar for each sample.

The taxonomic composition of broilers’ cecal microbiota revealed a predominance of the *Firmicutes* phylum, followed by *Bacteroidetes*, *Proteobacteria*, *Epsilonbacteraeota* and *Actinobacteria* phyla, regardless of the birds’ age and experimental group (Figure 4). Three families—*Lachnospiraceae*, *Ruminococcaceae* and *Bacteroidaceae*—predominated in the bacterial populations, with different relative abundance depending on experimental conditions (Figure 4). The complete taxonomic composition of cecal microbiota in each sample, including the relative abundance of OTUs, is presented in Table S2.

**Figure 5:**
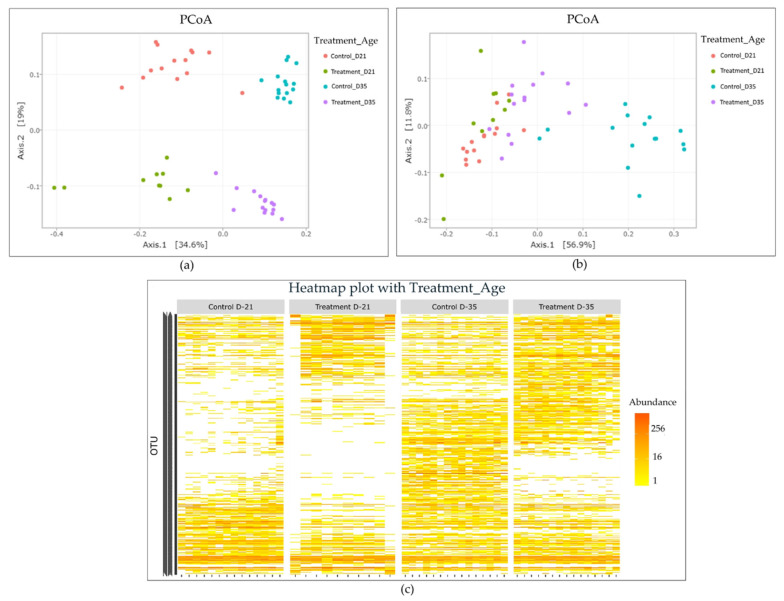
Representation of β-diversity of broilers’ cecal microbiota for the control and litter-treated groups on days 21 and 35. Principal coordinate analysis (PCoA) on samples from treated and control groups on days 21 and 35. (**a**) PCoA based on UniFrac distance (**b**) PCoA based on weighted UniFrac distance. (**c**) Community structure represented by a heatmap. The color scale represents the relative abundance of OTUs, yellow being the least and red the most abundant.

A principal coordinate analysis (PCoA) using UniFrac and weighted UniFrac (wUniFrac) distances was performed to visualize differences in microbial population structures among samples depending on treatment and age (Figure 5). A multivariate ANOVA (performed with Adonis) revealed a significant difference in microbial population structure among samples regarding age (*p* = 0.0001 for UniFrac and wUniFrac distance), experimental group (*p* = 0.0001 for UniFrac and wUniFrac distance), and the interaction of both variables (*p* = 0.0002 and *p* = 0.0001 for UniFrac and wUniFrac distance respectively). The broilers’ age explained about 29% and 33% of the total variance (for UniFrac and wUniFrac distance respectively). The litter treatment explained 19% and 12% of the total variance (for UniFrac and wUniFrac distance respectively), while the interaction of both variables explained 6% and 10% of the total variation (for UniFrac and wUniFrac distance respectively). Lastly, a higher segregation of samples according to their experimental group was observed using UniFrac distance than with wUniFrac distance (Figure 5), suggesting that the most abundant OTUs in cecal samples are phylogenetically closely related.

**Figure 6:**
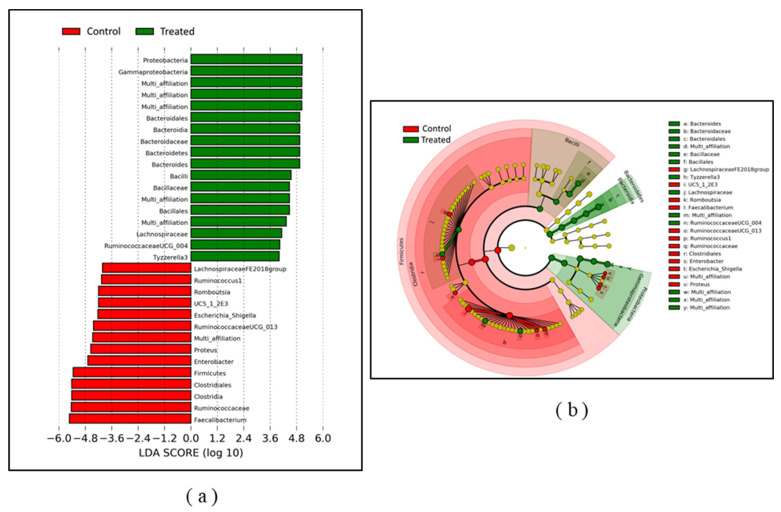
Bacterial taxa significantly differentiated broilers’ cecal communities from the litter-treated or control groups, identified by linear discrimination analysis coupled with effect size (LefSE). (**a**) Histogram of the LDA scores computed for taxa with different relative abundance depending on whether the ceca examined are from the litter-treated or the control group. Only taxa with a LDA threshold value > 3 are reported. (**b**) Cladogram presenting LefSE results of the identified taxa according to their phylogenetic characteristics.

A LEfSe analysis [21] was performed to identify OTUs showing a statistically different relative abundance between litter-treated and control groups at the end of the trial on day 35 (Figure 6). Taxa explaining the greatest statistical and biological differences between the two communities were identified in the histogram. *Bacteroidaceae*, *Bacillaceae* and *Lachnospiracea* families were significantly (*p* < 0.05) more abundant in broilers from the litter-treated group by day 35, while the *Ruminococcaceae* family was significantly (*p* < 0.05) more abundant in the control birds. Three genera—*Bacteroides*, *RuminococcaceaeUCG*_004, and *Tyzzerella3*—were significantly (*p* < 0.05) more abundant in litter-treated birds. In contrast, nine genera—including *Faecalibacterium*, *Enterobacter*, *Proteus*, *RuminococcaceaeUCG*_013, and *Escherichia*_*Shigella*—were significantly (*p* < 0.05) more abundant in control broilers on day 35.

## 3. Discussion

Numerous previous studies have focused on whether feed additives affect the colonization of broilers by *Campylobacter* [14,18,22]. However, fewer studies have explored the role of litter in *Campylobacter* perpetuation and transmission in broiler flocks, which was first investigated more than 30 years ago [23]. As *Campylobacter* contamination of broiler flocks is still a major issue in 2020, it is necessary to explore new control strategies to reduce the contamination level in broiler intestines, and applying a litter treatment could be a practical solution. In this study, the effect of a commercially marketed live bacterial complex registered as a biocide (No. 71001/040.03.001) in accordance with the European Directive 98/8/EC was tested on artificially inoculated broilers. Previous laboratory work demonstrated that culture supernatants of bacteria constituting the product were able to inhibit *Campylobacter* growth in vitro (data not shown). Moreover, Newell and Fearnley [24] reported that the dryness of fresh litter is considered lethal to *Campylobacter*, and there is an absorbent compound in the product tested. The bactericidal and absorbent properties of this commercial product are what led us to test it in vivo.

No effect of the litter treatment on *Campylobacter* counts was observed during the first trial, when each broiler was orally inoculated with 4 log_10_ CFU/g of *Campylobacter*. At the end of the trial, the *Campylobacter* counts in the ceca of broilers with treated litter were similar to those for the control group (on untreated litter). To check whether this challenge condition was too stringent and could have limited the effect of the litter treatment, another trial was performed to see whether *Campylobacter* transmission among broilers could be reduced in the litter-treated group by orally inoculating only one-third of the birds. This second trial highlighted the quick spread of *Campylobacter* among broilers, as they were all contaminated within five days. This finding is in agreement with the high transmission rate of *Campylobacter* in colonized flocks demonstrated in previous studies [25,26]. Despite application of the litter treatment every week since the beginning of rearing and the bactericidal activity of the product, it did not impact *Campylobacter* spread.

Gut microbiota in broilers develop immediately after hatching and are impacted by many factors related to the host and its environment [27]. Litter has been identified as an influencing factor, since broilers continuously ingest litter material and microorganisms [27]. Litter treatment did not affect broilers’ colonization by *C. jejuni* in this study. To check whether the litter treatment affected other bacterial taxa from broilers’ cecal microbiota, a 16S metabarcoding approach was used. Both alpha and beta diversity were affected by the litter treatment. In fact, a higher genetic diversity and lower richness were observed in broilers on treated litter on day 35 compared with control birds, suggesting fewer species with more evenly distributed relative abundance in the cecal microbiota of broilers on treated litter. While the treatment explained 12% to 19% of the total variations in microbial population structure, the age of broilers explained 29% and 33%, suggesting that broiler age had a higher impact on cecal microbial communities than the treatment of litter. While the impact of broiler age on cecal microbiota has already been reported [28,29], modifications to cecal microbiota due to the litter treatment could be the result of several phenomena. Indeed, the product could have a direct effect on cecal microbiota after the ingestion of litter material by broilers through the action of living bacteria included in the treatment (i.e., bacterial competition and gut colonization of the host). The treatment could also have an indirect effect through the modification of litter microbiota due to the action of absorbent compounds and the presence/development of living bacteria from the product in the litter itself, which is then ingested by the broilers. Indeed, the microbiota of the litter, which is frequently inoculated by poultry feces during the rearing period, are known to be influenced by environmental factors including pH, diet and moisture [30,31], so litter intake could in turn modify the composition of cecal microbiota. 

Different taxa explaining the greatest difference between microbial communities of litter-treated and control birds were identified. For example, the *Bacilli* class and the *Bacillaceae* family were discriminative for treated broilers. This could be the result of colonization of the broiler gut by *Bacillus amyloliquefaciens,* which is one of the bacterial species contained in the litter treatment product used during the in-vivo trial. However, the discriminatory power of 16S RNA metabarcoding is not sufficient to confirm this hypothesis, since the taxonomic affiliation of OTUs from the *Bacilli* class ended at family or genus level. It is also interesting to note that the *Escherichia*_*Shigella* genus and *Clostridia* class characterized the cecal microbiota of broilers from the control group. As several broiler pathogens belong to this genus or class (e.g., *Clostridium perfringens*, *Escherichia coli*), the impact of the litter treatment on these pathogens could be further investigated. In previous studies, the presence of *Faecalibacterium* [32] or increased abundance of the *Clostridiales* regrouping members of the *Clostridiaceae*, *Lachnospiraceae* and *Ruminococcacea* [33] have been associated to *Campylobacter* colonization. In our study, *Faecalibacterium*, *Clostridiales* and *Ruminococcacea* were strongly associated to the control group. Based on these results, it would have been expected that the control group harbored significantly more *Campylobacter* counts in ceca than the treated group, but this trend was not observed. On the other hand, Thibodeau et al. (2017) observed only slight modifications of the microbiota in response to *C. jejuni* infection and suggest that it acts as a super-colonizer due to its potential commensal lifestyle. Therefore, the modifications of the microbiota caused by the litter treatment could not be sufficient to reduce the colonization by *Campylobacter*. 

To conclude, the litter treatment did not reduce *Campylobacter* loads in broilers’ ceca as expected during these in-vivo trials. However, this study was conducted in a level 2 facility under strictly controlled conditions, which does not reflect the rearing environment of commercial flocks. As a consequence, a field study should be performed to test the product in poultry farm conditions. Although, in this study, the litter treatment was not successful in reducing *Campylobacter* colonization in broilers, it did modify the broilers’ cecal microbiota. Combining it with other products (feed or water additives) acting together synergistically could improve the reduction of broilers’ cecal colonization by *Campylobacter*.

## 4. Materials and Methods

### 4.1. In-Vivo Experimental Design

#### 4.1.1. *C. jejuni* Culture Condition

The strain used for the broiler challenge, *C. jejuni* C97Anses640, was isolated from a poultry product and characterized by multilocus sequence typing [34] as belonging to the ST-45 complex. The strain was stored at −70 °C in peptone broth containing 20% (*v*/*v*) glycerol. Five days before the birds were challenged, the strain was recovered from frozen stock after plating on mCCDA (selective modified charcoal cefoperazone deoxycholate agar) (Thermo Fisher Diagnostics, Dardilly, France) at 41.5 °C for 48 h under a microaerobic atmosphere (85% N_2_, 10% CO_2_ and 5% O_2_). One colony was used to inoculate 10 mL of brucella broth (BD Biosciences, San Jose, CA, USA). After 24 h at 41.5 °C in a microaerobic atmosphere, the bacterial suspension was diluted to 10^5^ CFU/mL in tryptone salt broth (BioMérieux, Bruz, France). 

#### 4.1.2. Poultry and Housing Conditions

Two independent trials were carried out at the Animal Biosafety Level 2 facilities of ANSES’s Ploufragan Laboratory in northwest France, an approved establishment for animal experimentation (No. C-22-745-1). It was conducted in accordance with the principles and specific guidelines presented in the Guide for the Care and Use of Agricultural Animals in Research and Teaching [35], and the protocol was approved by ethical committee ComEth Anses/ENVA/UPEC (11/06/13-3). Before the trial began, the experimental facilities (rooms, pens, feeding and drinking systems) were cleaned and disinfected. A total of 108 day-of-hatch Ross PM3 broilers (males and females) were included in the study for the two trials. Chicks vaccinated against infectious bronchitis were purchased from a commercial hatchery. Trials lasted 35 days from reception of the chicks to the last sampling procedure. Birds were kept in 3.42 m² (1.85 × 1.85 m) floor pens. Litter was composed of unused wood shavings. The facilities have programmable electric lights, automated electric heating and forced ventilation. There were eighteen hours of lighting per day throughout the experiment apart from for days 1 to 7, when there were 23 h of lighting per day. The environmental temperature was gradually reduced from 32 °C on day 1 to 18 °C on day 35. Chicks were randomly assigned to the two experimental groups (control and litter-treated) (cf. Section 4.1.3). Feed was weighed and manually distributed. The experimental diets were formulated and manufactured by a commercial feed meal company. They were fairly standard diets for broilers. The starter diet was offered to birds from day 1 to day 21, and the finisher diet from day 22 to day 35. Feed was presented as granulated crumbs. Feed and water were available ad libitum throughout the rearing period. On day 1 and at each sampling time, each bird was individually weighed, and feed intake per pen was recorded. Environmental swabs were taken from the facilities and transport crates and five randomly selected chicks were humanely euthanized (electronarcosis followed by bleeding) upon arrival in order to check the absence of *Campylobacter* colonization. Microbial analyses were carried out according to the ISO 10272–1:2017 standard [36]. 

#### 4.1.3. Chick Colonization Assay

**Figure 7:**
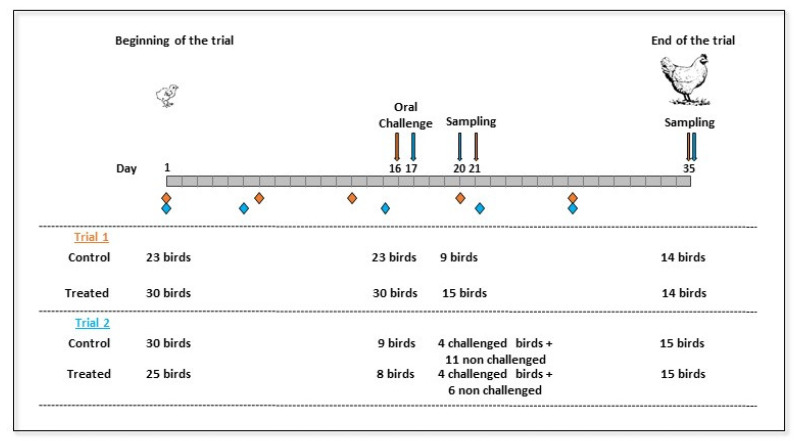
Diagram showing the main steps of the experimental design (with dates and number of broilers) used for the two in-vivo trials. Diamonds represent application of the litter treatment. Orange is used for the first trial and blue for the second one.

A similar protocol was carried out for the two trials, but several specifications regarding the number of birds per group, the day of interventions and the way the broilers were challenged were applied (Figure 7). Two groups of chicks were used for each trial: one for the control group and the other one for the litter-treated group. The litter treatment in powder form was spread manually on the litter in order to cover the whole surface of the pen before the chicks’ arrival (200 g per pen), and then once a week (100 g per pen) according to the manufacturer’s recommendations. On day 17 for the first trial (day 16 for the second one), all the birds in each group were orally challenged with 1 × 10^4^ CFU of *C. jejuni* C97ANSES640 in a 100 μL suspension of tryptone salt broth by oral gavage during the first trial. For the second trial, only one-third of the birds per group were challenged. For sampling purposes, the broilers were anesthetized by electronarcosis before being ethically euthanized by exsanguination for both trials. They were sampled on day 20 for the first trial (21 for the second one) and day 35 for both trials. During the second trial, four challenged birds were randomly selected from those sampled on day 21. Ceca were recovered for *Campylobacter* and microbiota analysis. During both trials, litter was sampled and tested for *Campylobacter* detection on days 10 (to check the absence of *Campylobacter* in chicks before inoculation), 20 or 21, and 35, according to the ISO 10272–1:2017 standard [36].

#### 4.1.4. Cecal Enumeration of *Campylobacter* spp.

*Campylobacter* enumerations were assessed in cecal contents after direct plating according to the decimal dilution method. Ceca were weighed and diluted 1:10 (*w*/*v*) in tryptone salt broth. After 1 min of homogenization in a stomacher (Interscience, Saint-Nom-la-Bretèche, France), 10-fold dilution series were carried out in tryptone salt broth and 50 μL of the dilutions was spread on mCCDA plates using an automatic easySpiral Dilute^®^ plater (Interscience, Saint-Nom-la-Bretèche, France). After 48 h of incubation at 41.5 °C under microaerobic conditions, colonies showing the morphology of *Campylobacter* were counted. The detection limit for enumeration of *Campylobacter* was 1 × 10^2^ CFU/g (2 log_10_ CFU/g) of cecal content. To conduct microbiota analyses, 1 mL of the first dilution of cecal content was centrifuged at 10,000× *g* for 10 min, then the supernatant was discarded and the remaining pellet was stored at −20 °C. 

#### 4.1.5. Statistical Analyses

The body weight and *Campylobacter* count in the ceca of broilers from the control and litter-treated groups on each sampling date were compared. Student’s parametric test was used when the normality and homogeneity criteria of the variances were validated; otherwise, the non-parametric Mann–Whitney test was used. A *p*-value < 0.05 (*p* < 0.05) was used to indicate whether differences were statistically significant. 

### 4.2. Cecal Microbiota Analyses

#### 4.2.1. DNA Extraction

Genomic DNA was extracted from frozen cecal pellets, obtained after centrifugation of 1 mL of diluted cecal content (1:10), using a NucleoMag Tissue Kit (Macherey-Nagel, Hoerdt, France). Briefly, pellets were resuspended with 500 µL of T1 lysis buffer (NucleoMag Tissue kit) then 100 mg of 0.1 mm Zirconia/Silica Beads (BioSpec, Bartlesville, CA, USA) was added to the suspensions. Mechanical lysis was next performed at a maximum intensity of 30 m/s for 3 min using the Star Beater (VWR, Fontenay-sous-Bois, France), followed by chemical lysis: the lysates were supplemented with 25 µL of Proteinase K (NucleoMag Tissue kit) then incubated at 70 °C for 30 min with regular vortexing. After centrifuging the samples for 2 min at 13,000× *g*, 225 µL of lysate supernatants was transferred into a standard 12-well plate and used for DNA extraction with a KingFisher Duo Prime instrument (Thermofisher Scientific, Illkirch-Graffenstaden, France). DNA concentrations were assessed using a Qubit 2.0 Fluorometer and Qubit dsDNA HS Assay Kit or Qubit dsDNA BR Assay Kit (Thermofisher Scientific, Illkirch-Graffenstaden, France). DNA extracts were stored at −20 °C until microbiota analyses.

#### 4.2.2. PCR Amplification of Bacterial 16S Ribosomal Genes and MiSeq Illumina Sequencing

To assess the microbial composition of broilers’ ceca, hypervariable region V3-V4 of the 16S rDNA was sequenced using Illumina technology. The V3-V4 region was amplified from genomic DNA using F343 and R784 primers (F343: CTTTCCCTACACGACGCTCTTCC GATC**TT**ACGGRAGGCAGCAG; R784: GGAGTTCAGACGTGTGCTCTTCCGATCTTA CCAGGGTATCTAATCCT) and 30 PCR cycles with an annealing temperature of 65 °C. A second PCR of 12 cycles was performed with 6-bp homemade index sequences added to the reverse primer for single multiplexing. The resulting PCR amplicons were purified and loaded onto the Illumina MiSeq cartridge according to the manufacturer’s recommendations (Illumina Inc., San Diego, CA, USA). Pair-end sequences were assembled using Flash software [37] with an overlap of at least 10 bp between the forward and reverse sequences and a mismatch no greater than 10%. The quality of sequences was checked using four bacterial samples that were run routinely in the sequencing facility at the same time as the current samples, and the run quality was internally checked using control libraries generated from the PhiX virus (Illumina PhiX control; Illumina Inc., San Diego, CA, USA). Paired sequences were assigned to their sample by the GeT-PlaGE platform (Toulouse, France), based on the previously integrated index. Sequencing reads were deposited in the National Center for Biotechnology Information’s Sequence Read Archive (Bioproject accession number: PRJNA625431).

#### 4.2.3. Sequence Analyses

Sequences were processed using FROGS, a galaxy-supported pipeline [38]. Briefly, sequences were cleaned by removing sequences with ambiguous bases, of an unexpected length (<380 or >500 nucleotides), or those without a primer sequence at both 3′- and 5′-ends (no mismatch allowed) before dereplication. After the sequences were clustered into OTUs using SWARM [39], and following FROGS guidelines [38], chimeras were removed using VSEARCH [40] combined with a cross-sample validation step [38]. OTUs were then filtered according to their size (OTUs with an abundance below 5 × 10^−5^ were removed) and the BLAST algorithm [41] was used for taxonomic assignment against the SILVA 16S database (version 132 filtered at a pintail score of 80) [42]. A phyloseq R package [43] object was generated to perform further statistical analysis.

#### 4.2.4. Statistical Analyses

Descriptive statistical analyses on the diversity and structure of cecal microbiota were performed using the phyloseq R package implemented in FROGS [38]. Shannon and InvSimpson diversity indices were calculated as well as the Chao1 richness estimator (Table S1) to describe microbiota diversity and richness. The effect of the litter treatment on these indices was investigated using ANOVA followed by Tukey tests, or a Kruskal–Wallis test followed by Mann–Whitney tests for data not satisfying parametric assumptions. Since broilers’ microbiota are known to be affected by their age [28,29], we also looked at the impact of the birds’ age and the interaction between treatment and age on microbiota diversity and structure. A UniFrac or wUniFrac distance matrix was calculated after data rarefaction, and plotted using multidimensional scaling (MDS) to investigate the structure of the bacterial community. An ADONIS pairwise test was used to check significance. To identify OTUs whose relative abundance were statistically different between litter-treated and control birds, the linear discriminant analysis (LDA) effect size (LEfSe) [21] was performed, and only OTUs with a LDA score over 3 were reported.

## Figures and Tables

**Table 1:** Body weight (mean ± SD in g) of broilers during *Campylobacter* colonization trials. The number of broilers (n) per group is in brackets.

	Trial 1		Trial 2	
	Day 20	Day 35	Day 21	Day 35
Control	824 ± 72 (n = 23)	2444 ± 286 (n = 14)	878 ± 145 (n = 29)	2350 ± 286 (n = 15)
Litter treatment	856 ± 71 (n = 30)	2497 ± 229 (n = 14)	880 ± 160 (n = 25)	2341 ± 345 (n = 15)

## Supplementary Materials

The following are available online at https://www.mdpi.com/2076-0817/9/5/333/s1, Table S1: Values of alpha diversity indices for all the broilers’ cecal samples; Table S2: Table of OTUs relative abundance and their taxonomic affiliation in broilers’ cecal samples.

## References

[1] EFSA (2019). Scientific report on the European Union One Health 2018 Zoonoses Report. EFSA J..

[2] Havelaar A.H., Ivarsson S., Lofdahl M., Nauta M.J. (2013). Estimating the true incidence of campylobacteriosis and salmonellosis in the European Union, 2009. Epidemiol. Infect..

[3] Van Cauteren D., De Valk H., Sommen C., King L.A., Jourdan-Da Silva N., Weill F.X., Le Hello S., Megraud F., Vaillant V., Desenclos J.C. (2015). Community Incidence of Campylobacteriosis and Nontyphoidal Salmonellosis, France, 2008–2013. Foodborne Pathog. Dis..

[4] Mughini Gras L., Smid J.H., Wagenaar J.A., de Boer A.G., Havelaar A.H., Friesema I.H., French N.P., Busani L., van Pelt W. (2012). Risk factors for campylobacteriosis of chicken, ruminant, and environmental origin: A combined case-control and source attribution analysis. PLoS ONE.

[5] Rosner B.M., Schielke A., Didelot X., Kops F., Breidenbach J., Willrich N., Golz G., Alter T., Stingl K., Josenhans C. (2017). A combined case-control and molecular source attribution study of human *Campylobacter* infections in Germany, 2011–2014. Sci. Rep..

[6] Thepault A., Rose V., Quesne S., Poezevara T., Beven V., Hirchaud E., Touzain F., Lucas P., Meric G., Mageiros L. (2018). Ruminant and chicken: Important sources of campylobacteriosis in France despite a variation of source attribution in 2009 and 2015. Sci. Rep..

[7] EFSA (2011). Scientific Opinion on *Campylobacter* in broiler meat production: Control options and performance objectives and/or targets at different stages of the food chain. EFSA J..

[8] EFSA_Panel_on_Biological_Hazards_(BIOHAZ) (2010). Scientific Opinion on Quantification of the risk posed by broiler meat to human campylobacteriosis in the EU. EFSA J..

[9] Hue O., Le Bouquin S., Laisney M.J., Allain V., Lalande F., Petetin I., Rouxel S., Quesne S., Gloaguen P.Y., Picherot M. (2010). Prevalence of and risk factors for Campylobacter spp. contamination of broiler chicken carcasses at the slaughterhouse. Food Microbiol..

[10] Hue O., Allain V., Laisney M.J., Le Bouquin S., Lalande F., Petetin I., Rouxel S., Quesne S., Gloaguen P.Y., Picherot M. (2011). *Campylobacter* contamination of broiler caeca and carcasses at the slaughterhouse and correlation with *Salmonella* contamination. Food Microbiol..

[11] Hansson I., Pudas N., Harbom B., Engvall E.O. (2010). Within-flock variations of *Campylobacter* loads in caeca and on carcasses from broilers. Int. J. Food Microbiol..

[12] (2017). The European Commission. COMMISSION REGULATION (EU) 2017/1495 of 23 August 2017 amending Regulation (EC) No 2073/2005 as regards Campylobacter in broiler carcases. Off. J. Eur. Union.

[13] Sibanda N., McKenna A., Richmond A., Ricke S.C., Callaway T., Stratakos A.C., Gundogdu O., Corcionivoschi N. (2018). A Review of the Effect of Management Practices on *Campylobacter* Prevalence in Poultry Farms. Front. Microbiol..

[14] Guyard-Nicodeme M., Keita A., Quesne S., Amelot M., Poezevara T., Le Berre B., Sanchez J., Vesseur P., Martin A., Medel P. (2016). Efficacy of feed additives against *Campylobacter* in live broilers during the entire rearing period. Poult. Sci..

[15] Gracia M.I., Millan C., Sanchez J., Guyard-Nicodeme M., Mayot J., Carre Y., Csorbai A., Chemaly M., Medel P. (2016). Efficacy of feed additives against *Campylobacter* in live broilers during the entire rearing period: Part B. Poult. Sci..

[16] Meunier M., Guyard-Nicodeme M., Dory D., Chemaly M. (2016). Control strategies against *Campylobacter* at the poultry production level: Biosecurity measures, feed additives and vaccination. J. Appl. Microbiol..

[17] Saint-Cyr M.J., Guyard-Nicodeme M., Messaoudi S., Chemaly M., Cappelier J.M., Dousset X., Haddad N. (2016). Recent Advances in Screening of Anti-*Campylobacter* Activity in Probiotics for Use in Poultry. Front. Microbiol..

[18] Saint-Cyr M.J., Haddad N., Taminiau B., Poezevara T., Quesne S., Amelot M., Daube G., Chemaly M., Dousset X., Guyard-Nicodeme M. (2017). Use of the potential probiotic strain *Lactobacillus salivarius* SMXD51 to control *Campylobacter jejuni* in broilers. Int. J. Food Microbiol..

[19] Guyard-Nicodeme M., Huneau-Salaun A., Tatone F.A., Skiba F., Quentin M., Quesne S., Poezevara T., Chemaly M. (2017). Effect of Feed Additives on Productivity and *Campylobacter* spp. Loads in Broilers Reared under Free Range Conditions. Front. Microbiol..

[20] Kassem I.I., Sanad Y., Gangaiah D., Lilburn M., Lejeune J., Rajashekara G. (2010). Use of bioluminescence imaging to monitor *Campylobacter* survival in chicken litter. J. Appl. Microbiol..

[21] Segata N., Izard J., Waldron L., Gevers D., Miropolsky L., Garrett W.S., Huttenhower C. (2011). Metagenomic biomarker discovery and explanation. Genome Biol..

[22] Thibodeau A., Letellier A., Yergeau E., Larriviere-Gauthier G., Fravalo P. (2017). Lack of Evidence That Selenium-Yeast Improves Chicken Health and Modulates the Caecal Microbiota in the Context of Colonization by *Campylobacter jejuni*. Front. Microbiol..

[23] Montrose M.S., Shane S.M., Harrington K.S. (1985). Role of litter in the transmission of *Campylobacter jejuni*. Avian Dis..

[24] Newell D.G., Fearnley C. (2003). Sources of *Campylobacter* colonization in broiler chickens. Appl. Environ. Microbiol..

[25] Shreeve J.E., Toszeghy M., Pattison M., Newell D.G. (2000). Sequential spread of *Campylobacter* infection in a multipen broiler house. Avian Dis..

[26] van Gerwe T., Miflin J.K., Templeton J.M., Bouma A., Wagenaar J.A., Jacobs-Reitsma W.F., Stegeman A., Klinkenberg D. (2009). Quantifying transmission of *Campylobacter jejuni* in commercial broiler flocks. Appl. Environ. Microbiol..

[27] Pan D., Yu Z. (2014). Intestinal microbiome of poultry and its interaction with host and diet. Gut Microbes.

[28] Awad W.A., Mann E., Dzieciol M., Hess C., Schmitz-Esser S., Wagner M., Hess M. (2016). Age-Related Differences in the Luminal and Mucosa-Associated Gut Microbiome of Broiler Chickens and Shifts Associated with *Campylobacter jejuni* Infection. Front. Cell. Infect. Microbiol..

[29] Kers J.G., Velkers F.C., Fischer E.A.J., Hermes G.D.A., Stegeman J.A., Smidt H. (2018). Host and Environmental Factors Affecting the Intestinal Microbiota in Chickens. Front. Microbiol..

[30] Lovanh N., Cook K.L., Rothrock M.J., Miles D.M., Sistani K. (2007). Spatial shifts in microbial population structure within poultry litter associated with physicochemical properties. Poult. Sci..

[31] Wang L., Lilburn M., Yu Z. (2016). Intestinal Microbiota of Broiler Chickens as Affected by Litter Management Regimens. Front. Microbiol..

[32] Thibodeau A., Fravalo P., Yergeau E., Arsenault J., Lahaye L., Letellier A. (2015). Chicken Caecal Microbiome Modifications Induced by *Campylobacter jejuni* Colonization and by a Non-Antibiotic Feed Additive. PLoS ONE.

[33] Connerton P.L., Richards P.J., Lafontaine G.M., O’Kane P.M., Ghaffar N., Cummings N.J., Smith D.L., Fish N.M., Connerton I.F. (2018). The effect of the timing of exposure to *Campylobacter jejuni* on the gut microbiome and inflammatory responses of broiler chickens. Microbiome.

[34] Dingle K.E., Colles F.M., Wareing D.R., Ure R., Fox A.J., Bolton F.E., Bootsma H.J., Willems R.J., Urwin R., Maiden M.C. (2001). Multilocus sequence typing system for *Campylobacter jejuni*. J. Clin. Microbiol..

[35] Federation_of_Animal_Science_Societies. Guide for the Care and Use of Agricultural Animals in Research and Teaching. www.fass.org/docs/agguide3rd/Ag_Guide_3rd_ed.pdf.

[36] (2017). International_Organization_for_Standardization. Microbiology of the food chain—Horizontal method for detection and enumeration of Campylobacter spp.—Part 1: Detection method (ISO 10272-1:2017). https://www.iso.org/standard/63225.html.

[37] Magoc T., Salzberg S.L. (2011). FLASH: Fast length adjustment of short reads to improve genome assemblies. Bioinformatics.

[38] Escudie F., Auer L., Bernard M., Mariadassou M., Cauquil L., Vidal K., Maman S., Hernandez-Raquet G., Combes S., Pascal G. (2018). FROGS: Find, Rapidly, OTUs with Galaxy Solution. Bioinformatics.

[39] Mahe F., Rognes T., Quince C., de Vargas C., Dunthorn M. (2014). Swarm: Robust and fast clustering method for amplicon-based studies. PeerJ.

[40] Rognes T., Flouri T., Nichols B., Quince C., Mahe F. (2016). VSEARCH: A versatile open source tool for metagenomics. PeerJ.

[41] Edgar R.C. (2010). Search and clustering orders of magnitude faster than BLAST. Bioinformatics.

[42] Quast C., Pruesse E., Yilmaz P., Gerken J., Schweer T., Yarza P., Peplies J., Glockner F.O. (2013). The SILVA ribosomal RNA gene database project: Improved data processing and web-based tools. Nucleic Acids Res..

[43] McMurdie P.J., Holmes S. (2013). phyloseq: An R package for reproducible interactive analysis and graphics of microbiome census data. PLoS ONE.

